# Supplementary figures and images for: Controlled temperature contrasts of three native and one highly invasive annual plant species in California
Source: PeerJ. 2025 Jan 21;13:e18794. doi: 10.7717/peerj.18794 (PMC11758916; doi:10.7717/peerj.18794)

## Bromus rubens

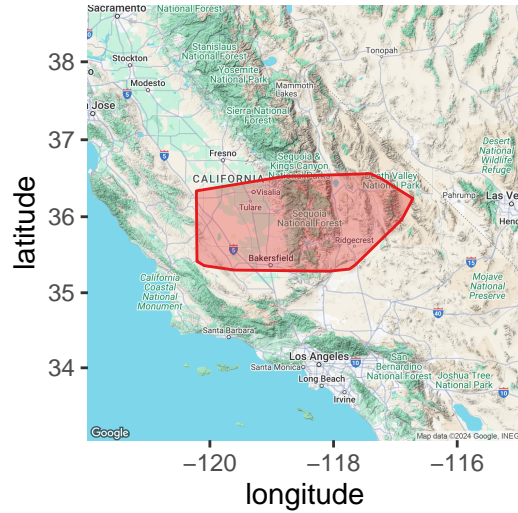

## Layia platyglossa

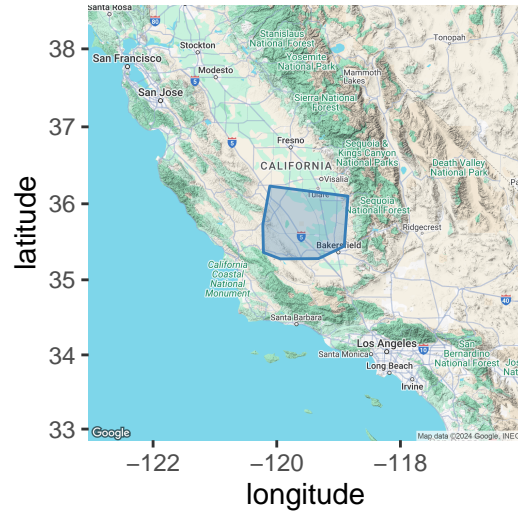

## Phacelia tanacetifolia

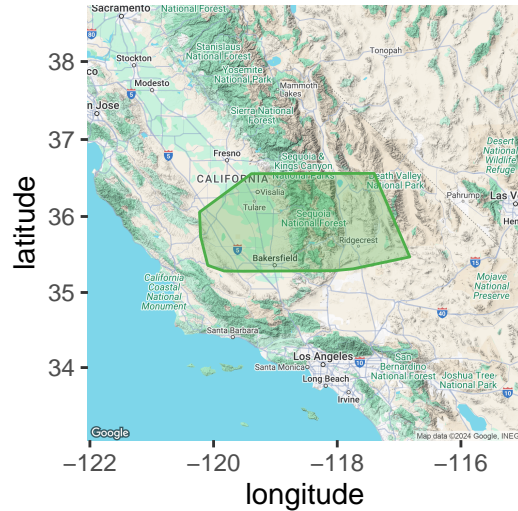

## Salvia columbariae

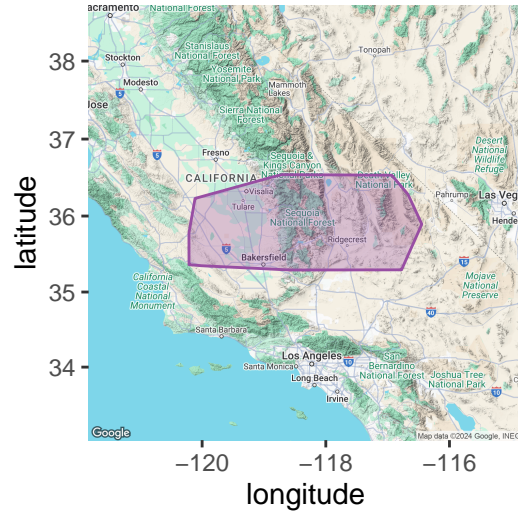

Supplement: Supplemental Information 1 — All species tested in the greenhouse trials were selected and reported observations mapped. Colours for each species are consistent through all figures. The map was generated utilizing the R package ggmaps and images of sites were taken via satellite imagery from Google Earth TM. [file peerj-13-18794-s001.pdf]
