# Supplementary material for: Controlled temperature contrasts of three native and one highly invasive annual plant species in California
Source: PeerJ. 2025 Jan 21;13:e18794. doi: 10.7717/peerj.18794 (PMC11758916; doi:10.7717/peerj.18794)
Supplement: Supplemental Information 2 — Regression analyses were done independently for each species with temperature data acquired from WorldClim. [file peerj-13-18794-s002.docx]

**Table S1:** Linear regression analyses of maximum temperature of the warmest month and mean temperature of warmest quarter with GBIF observations for three native and one invasive annual plant species. Regression analyses were done independently for each species with temperature data acquired from WorldClim.

| Factor | Species | R^2^ | Estimate | SE | DF | T-value | P-value |
| --- | --- | --- | --- | --- | --- | --- | --- |
| Max temperature of Warmest month | *Bromus rubens* | 0.018 | 0.045 | 0.049 | 1 | 0.908 | 0.368 |
|  | *Layia platyglossa* | 0.269 | -0.108 | 0.059 | 1 | -1.819 | 0.102 |
|  | *Phacelia tanacetifolia* | 0.061 | 0.105 | 0.072 | 1 | 1.459 | 0.154 |
|  | *Salvia columbariae* | 0.014 | 0.056 | 0.052 | 1 | 1.084 | 0.281 |
| Mean temperature of warmest quarter | *Bromus rubens* | 0.014 | 0.059 | 0.071 | 1 | 0.836 | 0.407 |
|  | *Layia platyglossa* | 0.099 | -0.078 | 0.078 | 1 | -0.997 | 0.345 |
|  | *Phacelia tanacetifolia* | 0.073 | 0.155 | 0.096 | 1 | 1.612 | 0.116 |
|  | *Salvia columbariae* | 0.015 | 0.065 | 0.059 | 1 | 1.082 | 0.283 |
